# Tomatine Displays Antitumor Potential in In Vitro Models of Metastatic Melanoma

**DOI:** 10.3390/ijms21155243

**Published:** 2020-07-23

**Authors:** Simona Serratì, Letizia Porcelli, Stefania Guida, Anna Ferretta, Rosa Maria Iacobazzi, Tiziana Cocco, Immacolata Maida, Gabriella Tamasi, Claudio Rossi, Michele Manganelli, Stefania Tommasi, Amalia Azzariti, Gabriella Guida

**Affiliations:** 1 Experimental Pharmacology Laboratory, IRCCS Istituto Tumori Giovanni Paolo II, 70124 Bari, Italy; s.serrati@oncologico.bari.it (S.S.); l.porcelli@oncologico.bari.it (L.P.); r.m.iacobazzi@oncologico.bari.it (R.M.I.); a.azzariti@oncologico.bari.it (A.A.); 2 Dermatology Unit, University of Modena and Reggio Emilia, 41121 Modena, Italy; drstefaniaguida@gmail.com; 3 Department of Basic Medical Sciences Neurosciences and Sense Organs, University of Bari, 70124 Bari, Italy; anna.ferretta@uniba.it (A.F.); tizianamaria.cocco@uniba.it (T.C.); immacolata.maida@uniba.it (I.M.); m.manganelli1991@gmail.com (M.M.); 4 Department of Biotechnology Chemistry and Pharmacology, University of Siena, 53100 Siena, Italy; gabriella.tamasi@unisi.it (G.T.); claudio.rossi@unisi.it (C.R.); 5 Molecular Diagnostics and Pharmacogenetics Unit, IRCCS Istituto Tumori Giovanni Paolo II, 70124 Bari, Italy; s.tommasi@oncologico.bari.it

**Keywords:** metastatic melanoma, tomatine, angiogenesis, apoptosis, autophagy

## Abstract

There is a growing interest in the cytotoxic effects of bioactive glycoalkaloids, such as α-tomatine on tumor cells. Here, for the first time, we determine the antitumor potential of tomatine, a mixture of α-tomatine and dehydrotomatine, in metastatic melanoma (MM) cell lines harboring different BRAF and MC1R variants. We performed cytotoxicity experiments and annexin-V/propidium iodide staining to assess the apoptotic/necrotic status of the cells. ER stress and autophagy markers were revealed by Western Blot, whereas antiangiogenic and vascular-disrupting effects were evaluated through a capillary tube formation assay on matrigel and by ELISA kit for VEGF release determination. Cell invasion was determined by a Boyden chamber matrigel assay. Tomatine reduced 50% of cell viability and induced a concentration-dependent increase of apoptotic cells in the range of 0.5–1 μM in terms of α-tomatine. The extent of apoptosis was more than two-fold higher in ^V600^BRAF-D184H/D184H MC1R cells than in BRAF wild-type cells and ^V600^BRAF-MC1R wild-type cell lines. Additionally, tomatine increased the LC3I/II autophagy marker, p-eIF2α, and p-Erk1/2 levels in BRAF wild-type cells. Notably, tomatine strongly reduced cell invasion and melanoma-dependent angiogenesis by reducing VEGF release and tumor-stimulating effects on capillary tube formation. Collectively, our findings support tomatine as a potential antitumor agent in MM.

## 1. Introduction

Malignant melanoma is an aggressive type of tumor that mainly occurs on the skin, and it is characterized by poor prognoses for patients with metastatic disease. Despite novel therapeutic approaches, the treatment of metastatic melanoma (MM) still remains challenging. Therefore, additional efforts should be made to find more effective strategies for such a disease. Many studies on molecular mechanisms underlying metastatic melanoma progression have highlighted the pivotal role of biochemical pathways involved in endoplasmic reticulum (ER) stress, autophagy, and translational reprogramming [1,2,3]. We previously demonstrated that the activation of such tumorigenic pathways was strictly related to the genetic status of BRAF and melanocortin 1 receptor (MC1R) [1,2,4,5]. In particular, the ^V600^BRAF mutation and its relation with the MC1R–cAMP–MITF–PGC–1α axis switches on a metabolic reprogramming in melanoma, leading to decreased OXPHOS (oxidative phosphorylation) activity and increased HIF-1α expression. Additionally, ER stress has been associated with variations in tumor angiogenesis [6,7], which is known to play a key role in cancer growth and invasion.

In the last few years, there has been a growing interest in the beneficial effects of plant compounds and also marine-derived fungi and marine red algae [8,9]. In particular, glycoalkaloids extracted from plants have been investigated for their pharmaceutical and toxicological properties [10,11]. Among these glycoalkaloids is tomatine, a 10:1 mixture of α-tomatine and dehydrotomatine extracted from tomatoes [12,13]. Tomatine inhibits the growth of many types of human cancer cells, such as colon, breast, leukemia, lung, and prostate [14], and it has the ability to enhance apoptosis of androgen-independent human prostate cancer when given in combination with chemotherapy [15,16]. Interestingly, tomatine has proven to activate caspase-independent apoptosis by inducing the translocation of AIF (apoptosis-inducing factor) from mitochondria to the nucleus, in a neuroblastoma cell line, by triggering the p-ERK/eIF2α branch of the unfolded protein response [17]. Additionally, tomatine has shown antibiotic, anti-inflammatory, antioxidative, cardiovascular, and immune-stimulating effects, although the mechanisms of these actions need further investigation [10,16]. Currently, no data are available regarding the effects of tomatine on metastatic melanoma.

Furthermore, recent reports have recognized naturally occurring plant-derived substances, such as alkaloids, as antitumor agents endowed with antiangiogenic properties [18]. No data are available on the antiangiogenic effect of tomatine. Here, we seek to evaluate the antitumor potential of tomatine through the evaluation of its effects on proliferation, cell invasion, and tumor-angiogenesis in metastatic melanoma cells with different genetic contexts, basal autophagy, and the activation status of the α subunit of translation initiation factor 2 (p-eIF2α).

## 2. Results

### 2.1. Tomatine Composition

To explore the relevance of the response to tomatine in MM models, commercial tomatine was preliminarily characterized by HPLC–ESI–QqQ–MS/MS analyses. Tomatine revealed a mixture of the two glycoalkaloids α-tomatine and dehydrotomatine, corresponding to 87.1 ± 1.6% and 13.0 ± 0.8%, respectively (Figure S1), according to [13]. The mixture of the two components and their ratio is comparable to that measured in tomatine extracts from tomato fruits and various vegetative plant tissues, for which the cytotoxic effect, evaluated in several types of cancer cells, has been ascribed to α-tomatine [10]. In our experiments, we utilized tomatine, which is the mixture of α-tomatine and dehydrotomatine, but since the active compound is α-tomatine, we reported the drug concentration of this glycoalkaloid, expressed in micromolar (µM).

### 2.2. Tomatine Displayed Antitumor Potential in MM Cell Lines

**Figure 1:**
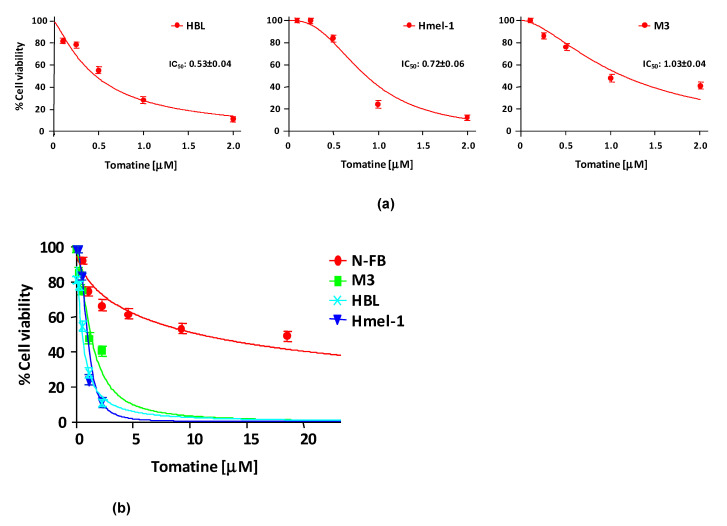
Cytotoxicity of tomatine in melanoma cell lines and in N-FB. (**a**) Cell viability (%)/dose plots of the mean of three different experiments ± SD conducted on HBL, hmel-1, and M3 melanoma cancer cells. In each experiment, every drug concentration (expressed in terms of α-tomatine) was repeated in six identical wells. The inserts in each graph report the IC_50_ values calculated using Calcusyn software. (**b**) Dot plot showing the dose–response curves of all tested cell lines, allowing for a comparison of cytotoxic effects.

In order to evaluate the cytotoxicity of tomatine, MM cell lines HBL, hmel-1, and M3 and normal fibroblast cell line N-FB were treated with increasing concentrations (expressed in terms of α-tomatine), as reported in Section 4. After 24 h of treatment, cell viability was reduced in a dose-dependent manner in all cell lines, allowing the determination of the concentration of α-tomatine yielding 50% inhibition of cell viability (IC_50_). The IC_50_ values were 0.53 ± 0.04, 0.72 ± 0.06, and 1.03 ± 0.04 µM for HBL, hmel1 and M3, respectively. Notably, tomatine was 10-fold less toxic in N-FB than MM cell lines since the IC_50_ value was 9.58 ± 0.6 μM. Dose–response plots, summarizing the data from three independent experiments performed in MM cell lines, are reported in panel A of Figure 1, whereas in panel B, the dot plot shows the dose–response curves of all tested cell lines, allowing for a comparison of cytotoxic effects.

**Figure 2:**
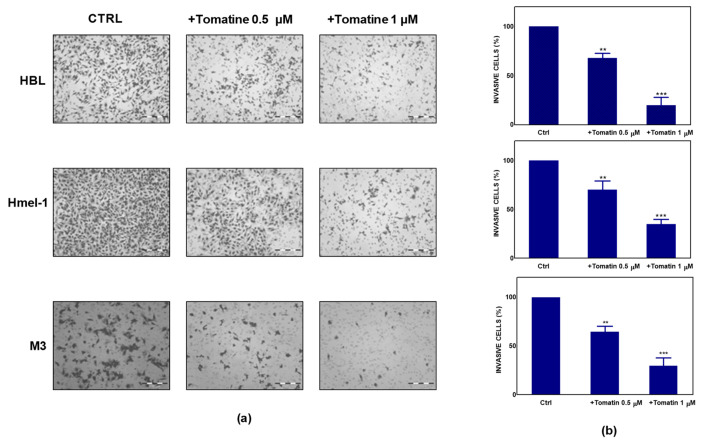
Effect of tomatine on melanoma cancer cell invasion. (**a**) Tomatine-dependent decrease of cancer cells invasion through matrigel-coated porous filters (magnification 50×). (**b**) Percentage of invasion in melanoma cells treated with tomatine (0.5–1 µM in terms of α-tomatine). Data are shown as mean ± SD of three different experiments performed in triplicate. ** *p* < 0.01 and *** *p* < 0.001, relative to vehicle-treated cells.

To further explore the antitumor potential of tomatine, we performed a Boyden chamber matrigel assay to analyze the effect of tomatine on cell invasion. As reported in Figure 2a, melanoma cells showed significant motility in a basal condition; conversely, the addition of tomatine considerably reduced the invasiveness of the cells. As quantified in Figure 2b, tomatine reduced the invasiveness by about 30–35% at the α-tomatine concentration of 0.5 µM and about 65–80% at 1 µM, suggesting the high inhibitory potential of tomatine on the invasive behavior of such MM cell models.

### 2.3. Tomatine Induced Cell Death through Apoptosis in Metastatic Melanoma Cells

**Figure 3:**
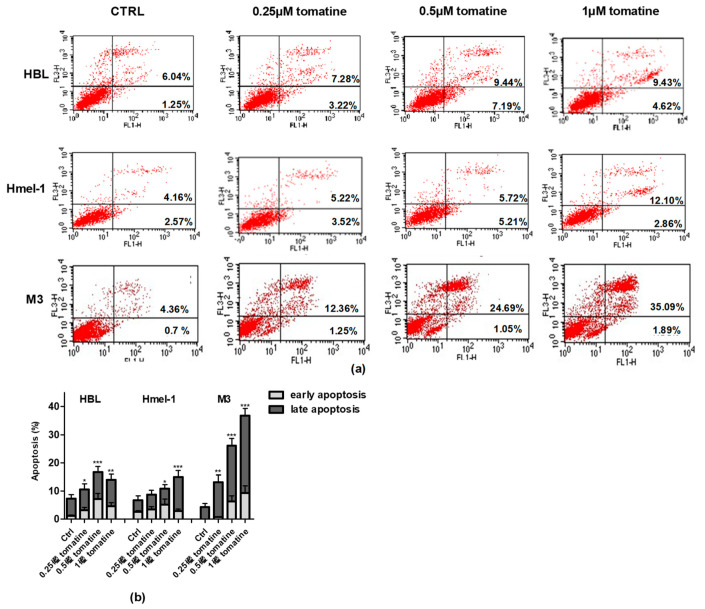
Apoptosis induced by tomatine in melanoma cell lines. HBL, hmel-1, and M3 cell lines were treated for 24 h with tomatine at 0.25, 0.5, and 1 µM (expressed as α-tomatine concentrations); afterwards, the cells were stained with annexin-V/PI and analyzed by FACS. (**a**) Representative analysis of annexin-V/PI stained cells; (**b**) histograms of the % of annexin-V (early apoptosis) and annexin-V/PI (late apoptosis)-positive cells in vehicle-treated cells and tomatine-treated ones. The results show the mean ± SD of three independent experiments. Data are shown as mean ± SD of three different experiments performed in triplicate. * *p* < 0.05, ** *p* < 0.01, and *** *p* < 0.001 of apoptosis (early + late), with respect to vehicle-treated cells.

To verify whether the cytotoxic effect was due to the induction of apoptosis, we performed annexin-V/PI staining of the cells, followed by flow cytometry analysis. For this purpose, we treated the MM cell line with tomatine at three different concentrations of α-tomatine (0.25, 0.5, and 1 µM) for 24 h. The results of the analysis, reported in Figure 3, showed that tomatine induced concentration-dependent apoptosis in all cells. However, in ^V600^BRAF M3 carrying a homozygous MC1R polymorphism, the percentage of annexin-V/PI positive cells was two-fold higher than in hmel-1 and HBL, in which tomatine induced comparable levels of apoptosis. Further evaluations, performed after 48 h, confirmed M3 as the most sensitive cell line to the proapoptotic effect of tomatine since the percentage of apoptotic cells increased nearly five-fold at 1 µM α-tomatine versus vehicle-treated cells (data reported in Figure S2).

### 2.4. Tomatine Induced Autophagy in BRAF Wild-Type Cell Line

**Figure 4:**
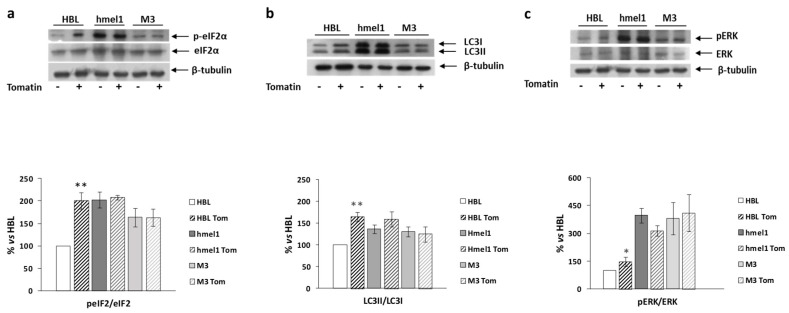
The effect of tomatine on the activation/expression levels of p-eIF2α and LC3 II/I, respectively, and on ERK phosphorylation levels in hmel-1 and M3 cells compared to BRAFwt HBL cells. (**a**) Representative Western blot of eIF2α and p-eIF2α levels, performed on whole cell lysates. Bar graph shows the p-eIF2α/eIF2α ratio calculated by densitometric analysis of the protein bands, normalized to tubulin, used as loading control. (**b**) Representative Western blot of LC3-I and LC3-II levels, performed on whole cell lysates. Bar graph shows the LC-II/LC-I ratio calculated by densitometric analysis of the protein bands, normalized to tubulin, used as loading control. (**c**) Representative Western blot analyses of pERK and ERK levels on whole cell lysates. Bar graph shows the pERK/ERK ratio, calculated by densitometric analysis of the protein bands, normalized to tubulin, used as loading control. Values are the mean ± SEM of three independent experiments, expressed as a percentage of the HBL value. Significance was calculated with Student’s test; * *p* < 0.05, ** *p* < 0.005 vs. vehicle-treated cells.

We previously reported that high levels of autophagy and chronic ER stress were strictly associated with a ^V600^BRAF genetic context in MM cells [1]. Hence, we investigated the effects of tomatine on the activation/expression levels of p-eIF2α and LC3 II/I in hmel-1 and M3 cell lines, compared to BRAFwt HBL cells. For this purpose, Western blotting analysis was performed on protein extracts from all cell lines treated with tomatine at 1 µM in terms of α-tomatine versus vehicle-treated ones. We found that tomatine induced a significant increase in p-eIF2α levels only in HBL cells (Figure 4a). No effect was found in other cell lines. According to these results, we also found an increased LC3II/I ratio in only the BRAFwt cell line (HBL), while no significant variations in both p-eIF2α and LC3II/I levels were observed in hmel-1 and M3 cell lines (Figure 4b). Furthermore, as also reported in previous research, high levels of autophagy and ERK phosphorylation were strictly associated to a ^V600^BRAF genetic context [1]. We investigated the effect of tomatine on ERK1/2 activation to understand if the tomatine-dependent increase of autophagy and p-eIF2α levels could be associated with increased phosphorylation of ERK1/2. Western blot analysis showed a significant increase of p-ERK levels after treatment with 1 μM tomatine in HBL cells, whereas no significant variations were observed in hmel-1 and M3 cell lines (Figure 4c). Collectively, these results suggest that tomatine induced ER stress, autophagy, and apoptosis in the HBL cell line with BRAFwt status.

### 2.5. Tomatine Displayed Antiangiogenic and Vascular Disrupting Effect

**Figure 5:**
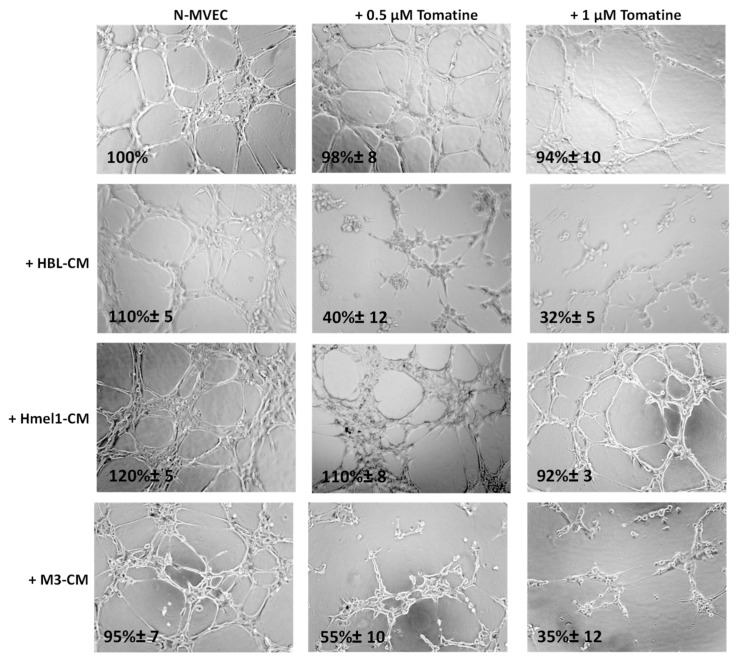
Effect of tomatine on melanoma-dependent vascular disruption of N-MVECs (microvascular endothelial cells, human dermal microvascular endothelial cells). Cells (12 × 10^3^) were plated on 10–12 mg/mL matrigel-coated tissue culture wells, treated immediately with melanoma cells CM, and after 3 h with tomatine (0.5 and 1 μM in terms of α-tomatine). The cells were photographed 9 h after plating. Six to nine photographic fields from three plates were scanned for each point, considering as 100% the area occupied by untreated N-MVECs. The results shown are representative of three different experiments.

The ability of N-MVECs (microvascular endothelial cells, human dermal microvascular endothelial cells) to move within ECM (extracellular matrix) is the basic requirement in the formation of ordered cords of endothelial cells. The final angiogenic event requires N-MVEC to organize in the interconnection of tubular structures appointed to give origin to blood vessels. We studied capillary tube formation on matrigel. In this assay, N-MVEC produced elongated processes that resulted in the formation of anastomosing cords of cells resembling a capillary plexus [19]. We investigated the effects of tomatine in melanoma-dependent angiogenesis in a vascular-disrupting assay and a neoangiogenesis assay, as described in the Material and Methods section. The addition of tomatine (0.5 and 1 μM in terms of α-tomatine) to the newly formed capillary-like structures, after 3 h incubation with melanoma cell conditioned media (CM), disrupted the network in the presence of HBL and M3 CM, and this effect increased over the following 9 h (data not shown). In contrast, hmel-1–CM seemed to protect the vessels from the vascular disruption activity of tomatine (Figure 5).

**Figure 6:**
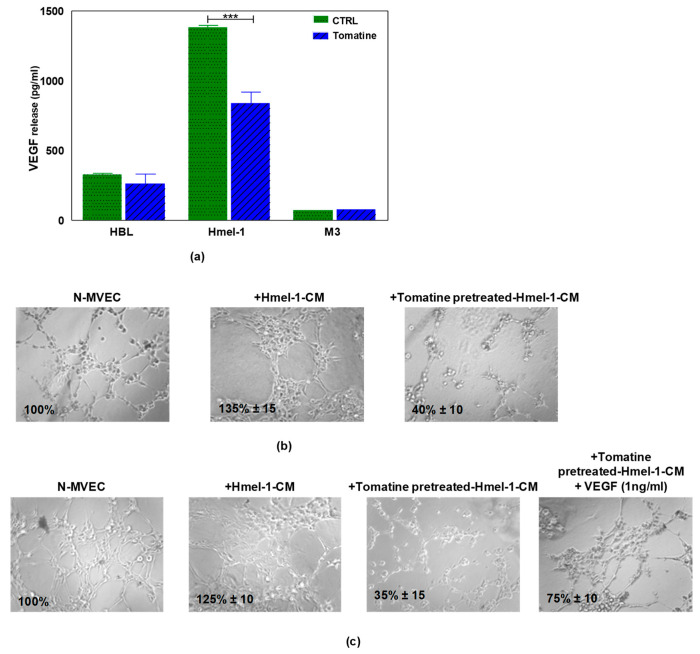
Effect of tomatine on VEGF release by metastatic melanoma (MM) cell lines and capillary morphogenesis in N-MVECs. (**a**) The graph displays the VEGF release by HBL, hmel-1, and M3 cells in basal conditions and after tomatine (1 μM in terms of α-tomatine) treatment. Values are means ± SD of three independent experiments; significance was calculated with Student’s *t*-test; *** *p* < 0.001 significantly different from basal control. (**b**) Effect of conditioned medium of hmel-1 and tomatine-pretreated hmel-1–CM, with or without external VEGF. (**c**) On capillary morphogenesis of N-MVECs.

Since the treatment of N-MVECs with hmel-1–CM stimulated neoangiogenesis and protected endothelial cells from tomatine inhibitory effects, we evaluated the release of VEGF by tumor cells before and after tomatine treatment. The data reported in Figure 6a demonstrated that hmel-1 released the highest amount of VEGF with respect to HBL and M3 cells (1383.41 vs. 326.98 and 73.38 pg/mL). Furthermore, the addition of tomatine to tumor cell lines drastically inhibited the release of the proangiogenic factor from hmel-1 cells with regards to HBL and M3 (837.25 vs. 261.91 and 75.59 pg/mL). Based on these results, a new capillary morphogenesis assay was performed in the presence of hmel-1–CM from cells pretreated with 1 μM in terms of α-tomatine for 24 h. As shown in Figure 6b, the proangiogenic effect shown in N-MVECs in the presence of hmel-1–CM was drastically reduced with the tomatine-pretreated hmel-1–CM. In order to evaluate whether the effect of tomatine is closely linked to the tumor-derived VEGF, we carried out a capillary morphogenesis assay by adding external VEGF (Figure 6c) to N-MVECs in the experimental conditions previously described. The angiogenetic ability of N-MVECs was partially restored by adding VEGF to pretreated hmel-1–CM, which revealed a correlation between the effects of tomatine treatment and the VEGF released by hmel-1.

**Figure 7:**
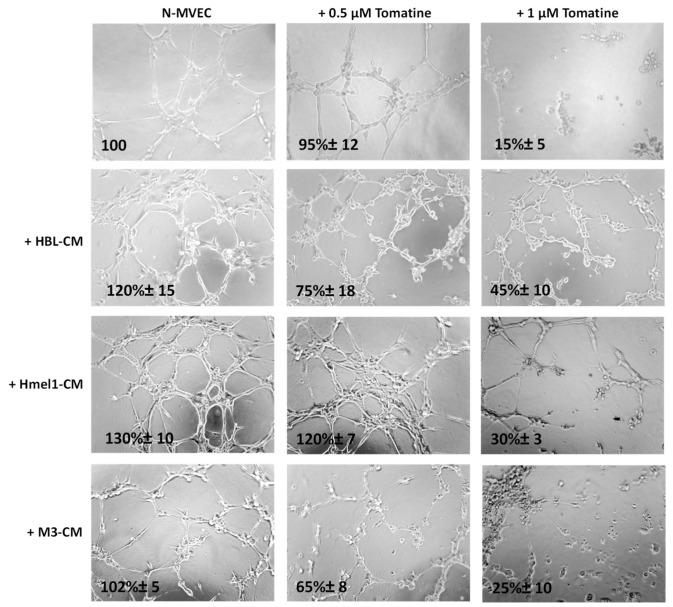
Effect of tomatine on melanoma-dependent neoangiogenesis of N-MVECs. Cells were plated on matrigel-coated tissue culture wells and treated with melanoma cell CM in the presence or absence of tomatine (0.5 and 1 µM expressed as α-tomatine concentrations). The cells were photographed 6 h after plating. The results shown are representative of three different experiments.

We further evaluated the tomatine effects on tumor neoangiogenesis. Figure 7 shows that after 6 h from plating on matrigel, N-MVECs produced networks of branching cords of cells. The ability to form a stable and tight network can be strengthened by the addition of melanoma cell CM. In particular, a stronger increase of the capillary formation (130% ± 10%) was observed in the presence of CM from a highly MM hmel-1 cell line; the phenomenon was minor (120% ± 15%) in HBL–CM and it was almost absent in M3–CM. In the presence of 0.5 μM in terms of α-tomatine, the neoangiogenesis was not affected by hmel-1–CM, while it decreased about 30–40% in the presence of HBL–CM and M3–CM, suggesting less proangiogenic properties of the latter compared to the hmel-1 cell line.

Interestingly, we observed a substantial reduction of neoangiogenesis when we used 1 µM tomatine in all the conditions, demonstrating the notable effect of this compound on neoangiogenesis.

## 3. Discussion

Several studies have indicated that α-tomatine inhibits the growth of various human cancer cell lines within 1–48 h in a wide range of concentrations [20,21,22,23,24,25]. Our results show that commercial tomatine inhibited both the cell invasion and cell viability of BRAFwt and ^V600^BRAF metastatic melanoma cells within the range of 0.5–1 μM (expressed as α-tomatine concentration) after 24 h treatment and highlight a previously unknown ability of the bioactive compound as an antiangiogenic and vasculature-disrupting agent that is far less toxic to normal fibroblasts. We found that tomatine induced an autophagic phenotype in BRAFwt cells, similar to that displayed in basal conditions by ^V600^BRAF cell lines. It is noteworthy that together with the autophagic phenotype, BRAFwt cells (HBL) treated with tomatine showed similar levels of ER stress marker p-eIF2α to those of hmel1 and M3 at the basal level, together with an increase of p-ERK1/2 level, thus suggesting that these conditions are a prerequisite for cell death induction by tomatine in such cells. ER stress is an adaptive response of cells, occurring when ER fails to cope with the excess of misfolded/unfolded protein loads, and it activates the unfolded protein response (UPR). The activation of this pathway may drive back the system to the former condition through the activation of autophagy, or alternatively, may result in apoptotic cell death during excessive levels of ER stress [26]. If the ER stress is not tolerable, cells undergo apoptosis, and the UPR promotes the phosphorylation of the α subunit of translation initiation factor 2 (eIF2α), generating a general attenuation of translational processes [27]. Therefore, we can speculate that the activation of the ER stress response led to an autophagic phenotype and a general attenuation of translational processes, with a consequent reduction of cell proliferation and apoptosis induction in the HBL cell line.

Instead, the physiological levels of ER stress and ERK1/2 activation and autophagy were enough to shift the balance towards apoptosis in the other cell lines, and perhaps, the apoptosis expanded in M3 from prolonged ER stress response activation in these cells. Accordingly, Wang at al. [25] reported that α-tomatine induced ER stress and triggered both caspase-dependent and caspase-independent apoptosis. Additionally, several authors recently reported a correlation between cellular stress response, translational reprogramming, and autophagy and apoptosis induction in the presence of a persistent upstream stress condition [28,29,30].

Translational control during endoplasmic reticulum stress plays a crucial role in tumor-dependent angiogenic processes. In basal conditions, hypoxia is associated with an increase in HIF-1α levels and a consequent increase of VEGF expression [31]. However, an upregulation of VEGF is also induced by ER stress [32]. The translation of VEGF [33], as well as of genes implicated in autophagy [34], is regulated by eIF2α phosphorylation. As a consequence, p-eIF2α induces an increase in nutrient supply by stimulating angiogenesis and recycling organelles. Accordingly, we found that hmel1 cells, which displayed a high basal level of p-eIF2α and HIF-1α, as reported in our previous characterization of such cells [1], released the highest amount of VEGF compared to other cell lines. Therefore, we can speculate that in hmel1, as a result of a basal condition of ER stress, a high physiological level of p-eIF2α sustained the release of VEGF, which was strongly reduced by tomatine. Hence, the principal effect of tomatine in the hmel1 cell line may be related to the inhibition of VEGF release by cells, as demonstrated by the VEGF-restoring effect on capillary tube formation in the specific assay conducted by adding external VEGF to N-MVECs in the presence of hmel1-pretreated tomatine-derived CM. The CM from other cell lines was less protective against the tomatine antiangiogenic effect, perhaps because these cells released less VEGF than hmel1; other proangiogenic factors may be involved. Aside from the need to confirm our in-vitro results in corresponding in vivo models, collectively, our findings show that tomatine inhibits cell viability as well as the invasive behavior of MM cell lines, and, more importantly, displays valuable antiangiogenic and vascular disrupting effects only in experimental conditions that mimic a proangiogenic tumor microenvironment, perhaps by affecting the ER/p-eIF2α/VEGF axis of tumor angiogenesis.

## 4. Materials and Methods

### 4.1. Tomatine: HPLC of Chemical Compounds

Tomatine (>75%) was purchased from TCI Chemicals Europe (Zwijndrecht, Belgium). Its actual composition was determined by reverse-phase liquid chromatography, using an HPLC Agilent 1200 Series system (Agilent Technologies, Milano, Italy) coupled with a TSQ Quantum Access mass spectrometer (Thermo Scientific, Milano, Italy), equipped with Electro-Spray ion source (ESI) and triple quadrupole analyzer (QqQ), employing the method previously described by Tamasi et al. [13].

### 4.2. Cell Culture

In the current study, three melanoma cell lines (hmel-1, M3, HBL) were utilized. Hmel-1 and M3 were extracted from skin metastases obtained from human sporadic melanoma biopsy specimens after the informed consent of patients. All cell lines were genotyped for NRAS BRAF and MC1R (Table 1), as described in [1,35,36]. HBL melanoma cells (BRAFwt) were a gift from Prof. G. Ghanem, Université de Bruxelles, Belgium, which were used as a control for all experiments. In addition, endothelial cells were studied. In detail, N-MVECs (microvascular endothelial cells, human dermal microvascular endothelial cells) were purchased from Lonza (Basel, Switzerland). Cell culture media used for MM cells was high-glucose Dulbecco’s modified Eagle’s medium (DMEM) supplemented with 10% (*v*/*v*) fetal bovine serum (FBS), 1% (*v*/*v*) l-glutamine, and 1% (*v*/*v*) penicillin/streptomycin at 37 °C in a humidified atmosphere at 5% CO_2_. All materials for cell culturing were purchased from EuroClone, Italy. The EGMTM-2 Endothelial Growth Medium-2 BulletKit^TM^ from Lonza, Switzerland, was used for N-MVEC cell culture. N-FB (normal human dermal fibroblasts, Lonza, Switzerland) were grown in an FGM-2 BulletKit (Lonza, Switzerland) supplemented with 2% FBS.

### 4.3. Cytotoxic Assay

Cells were seeded in 96-well culture plates at a density of 5000 cells/well. After 24 h, the culture medium was replaced with fresh medium (100 µL), with tomatine at increasing concentrations ranging between 0.12 and 2.38 mg/L (corresponding to 0.1–2 µM of α-tomatine; Figure 1). Then, cells were further incubated for 24 h and cell viability was assessed by MTT assay, as previously described [37]. Results were expressed as IC_50_ (the half-maximal inhibitory concentration of α-tomatine) or as % cell viability at the tested doses and were reported as dose–response curves using Calcusyn software, which allows IC_50_ determination.

### 4.4. Invasion Assay

A Boyden chamber was used to evaluate invasive ability through matrigel-coated porous filters, as described by [38]. Briefly, 50 mL of cell suspension (8 × 10^3^ cells) was placed in the upper compartment of the chamber, and migration was allowed to occur for 6 h. For assessment of the invasion, tomatine (0.5 and 1 µM in terms of α-tomatine) were dissolved in culture medium and placed in the upper and lower wells. The filter was removed and fixed in methanol. Nonmigrating cells on the upper surface of the filter were removed with a cotton swab, while migrated cells, adhered on the lower filter surface (pore-size 8 µm), were stained with Diff-Quick (Mertz-Dade AG, Dade International, Milan, Italy) and counted using a light microscope (OLYMPUS CKX41) on the whole migration surface per well. Mobilization was measured by the number of cells moving across the filter. Experiments were performed in triplicate. Migration was expressed as the mean ± standard deviation (SD) of the percentage of basal response. Data were indicated with * *p* < 0.01, and ** *p* < 0.001 compared to relative control.

### 4.5. Apoptosis Assay

The annexin-V–propidium iodide (PI) staining apoptosis test [36] was performed on HBL, hmel-1, and M3 cells after 24 h of incubation, with tomatine in the range of 0.25–1 µM in terms of α-tomatine. The cells were treated according to the instructions provided by the manufacturer. Briefly, after incubation time (24 h) at 37 °C and 5% CO_2_, cells were washed with PBS, suspended in binding buffer and then added with annexin-V FITC and PI for 20 min. The samples were then read by a flow cytometer, FACScan (Becton Dickinson, Milano, Italy).

### 4.6. Western Blot Analysis

Cells were suspended in hypotonic medium supplemented with an antiproteases cocktail tablet (Roche, Basel, Switzerland) and freeze-thawed three times to obtain total proteins. Western blotting analysis was performed, essentially as described by Ferretta et al. [39]. Total protein content was measured by the Bradford method, and 35 μg was electrophoretically separated on 10% acrylamide gel (Tris-Tricine SDS-PAGE) and transferred to the nitrocellulose membranes of a Trans-Blot^®^ Turbo™ Transfer Pack (#1704158, Bio-Rad, Segrate/Milano, Italy). Blots were blocked for 2 h in blocking buffer (PBS buffer, pH 7.4, with 0.2% Tween 20 and 5% nonfat milk) and incubated with primary antibody. Western blotting analysis of ERK/pERK was as described in Zanna et al. 2013 [35], and phosphorylated peIF2α/eIF2α was performed as described in Maida et al. (2019) [2]. The polyclonal antibodies, anti-eIF2α, and anti-peIF2α Ser51 were purchased from Cell Signaling Technology, Danvers, MA, USA [35]. The lipid-conjugated form of LC3 (LC3 II) localizes to the membranes of autophagosomes, and it can be separated from the nonconjugated form (LC3 I) by immuno-blotting since LC3 II migrates faster than LC3 I due to the extreme hydrophobicity of LC3 II. For LC3 detection, total cell proteins (30 μg) were separated on 12% Tris-Glycine SDS–PAGE and transferred onto nitrocellulose membranes. Western blot analysis was performed using a specific antibody against LC3B (Cell Signaling Technology). The secondary antibody was anti-rabbit HPR and anti-mouse HR (Millipore, Milano, Italy). Protein was detected by the chemiluminescent detection system (Bio-Rad), and the signal was quantified by densitometric analysis using the Chemidoc imaging system (Bio-Rad). The band intensity was quantitatively determined by densitometric analysis using Image Lab Software 5.2.1 (Bio-Rad Laboratories, Segrate/Milano Italy), and protein level intensity was normalized to tubulin expression. (Figure S3)

### 4.7. Angiogenesis and Vascular Disruption Assays

The ability of N-MVECs to move within ECM is the basic requirement to form ordered cords of endothelial cells. The final angiogenic event requires N-MVECs to organize in interconnecting tubular structures appointed to give origin to blood vessels. We have studied the capillary tube formation on matrigel. In this assay, N-MVECs produce elongated processes that eventuate in the formation of anastomosing cords of cells resembling a capillary plexus.

Matrigel (50 μL; 10–12 mg/mL) was pipetted into 0.64 cm (diameter) tissue culture wells and polymerized for 30 min to 1 h at 37 °C, as described in [40]. N-MVECs were plated (12 × 103/mL) in EGM-2 (endothelial growth medium, EGM™-2MV BulletKit™ Lonza, Switzerland) and 2% FBS. Capillary morphogenesis was performed in the presence of hmel-1- and M3-conditioned medium and tomatine (0.5–1 μM in terms of α-tomatine). Vascular disruption was evaluated in the same conditions as capillary morphogenesis, adding tomatine after three hours from cell seeding in the presence of the conditioned medium. The effects on morphogenesis and vascular disruption of endothelial cells were recorded after 6 h with an inverted microscope. Results were quantified at 6 h by measuring the percent field occupancy of the capillary projections. Six to nine photographic fields from three plates were scanned for each point.

### 4.8. Analysis of VEGF Release

Cells were incubated with or without 1 μM tomatine, and cell supernatant from each sample was utilized for the quantification of VEGF release by the Quantikine Human VEGF immunoassay (R&D Systems Minneapolis, MN, USA), as described in [41].

## Figures and Tables

**Table 1:** Genotype of melanoma cell lines analyzed.

Cell Line	Extracted from	MC1R	NRAS	BRAF Exon 15
HBL	metastasis	wt/wt	wt/wt	wt/wt
Hmel-1	metastasis	wt/wt	wt/wt	V600 K/wt
M3	metastasis	D184H/D184H	wt/wt	V600E/V600E

## Supplementary Materials

The following are available online at https://www.mdpi.com/1422-0067/21/15/5243/s1, Figure S1. Multiple Reaction Monitoring (MRM) chromatogram (via positive ion HPLC-ESI-QqQ-MS/MS analysis) of the commercial tomatine showing the presence of α-tomatine and dehydrotomatine. Figure S2: Analysis of Apoptosis after 48 h. Figure S3: Western blot images preformed on whole cell lysates and densitometric analysis of protein expression.
