# Supplementary material for: Tomatine Displays Antitumor Potential in In Vitro Models of Metastatic Melanoma
Source: Int J Mol Sci. 2020 Jul 23;21(15):5243. doi: 10.3390/ijms21155243 (PMC7432453; doi:10.3390/ijms21155243)
Supplement: Supplementary file 1 [file ijms-21-05243-s001.zip › suppl fig3 final.pptx]

## Slide 1
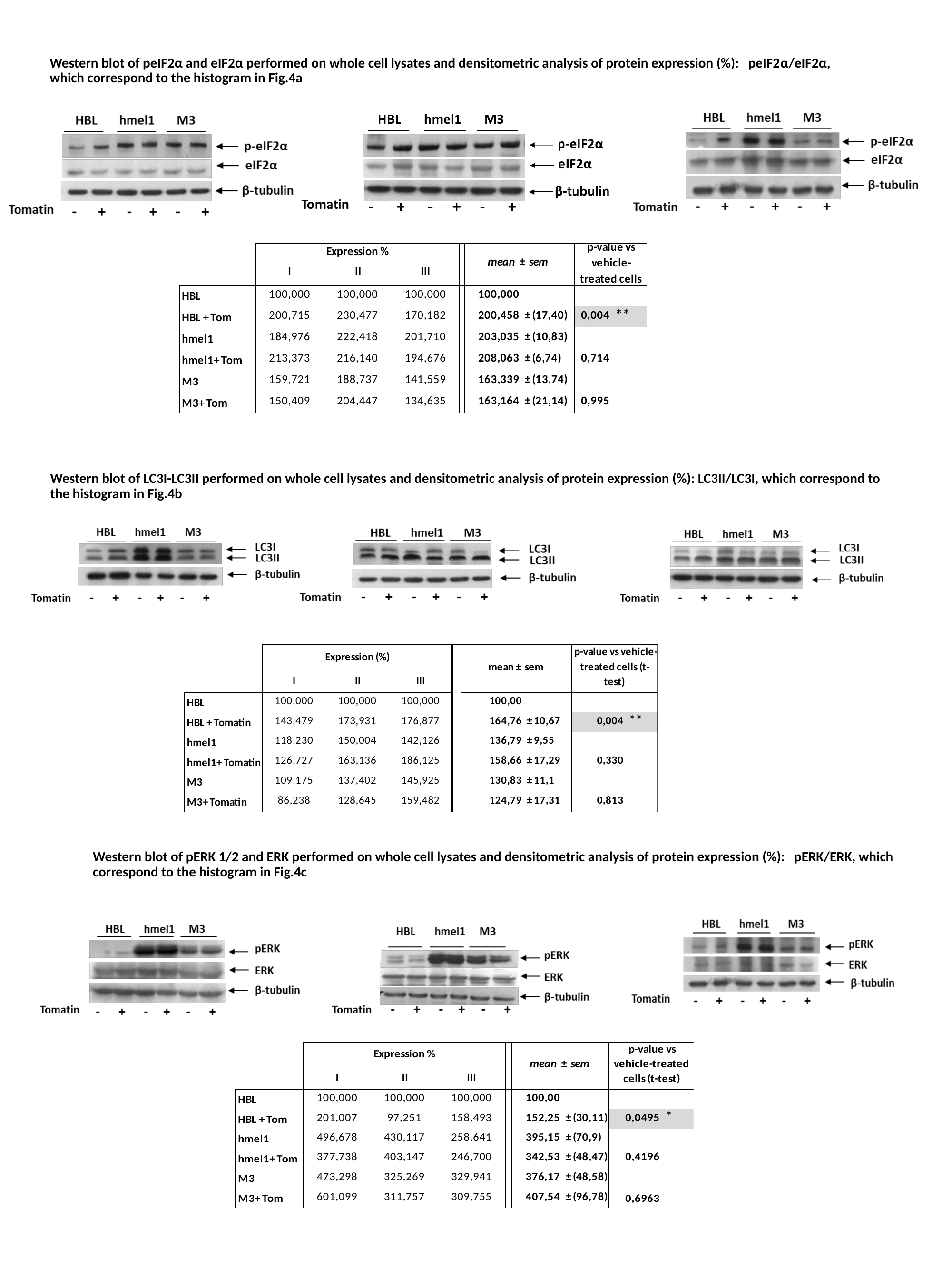

Western blot of peIF2α and eIF2α performed on whole cell lysates and densitometric analysis of protein expression (%): peIF2α/eIF2α, which correspond to the histogram in Fig.4a
Western blot of LC3I-LC3II performed on whole cell lysates and densitometric analysis of protein expression (%): LC3II/LC3I, which correspond to the histogram in Fig.4b
Western blot of pERK 1/2 and ERK performed on whole cell lysates and densitometric analysis of protein expression (%): pERK/ERK, which correspond to the histogram in Fig.4c
