# Supplementary figures and images for: Tomatine Displays Antitumor Potential in In Vitro Models of Metastatic Melanoma
Source: Int J Mol Sci. 2020 Jul 23;21(15):5243. doi: 10.3390/ijms21155243 (PMC7432453; doi:10.3390/ijms21155243)

## Slide 1
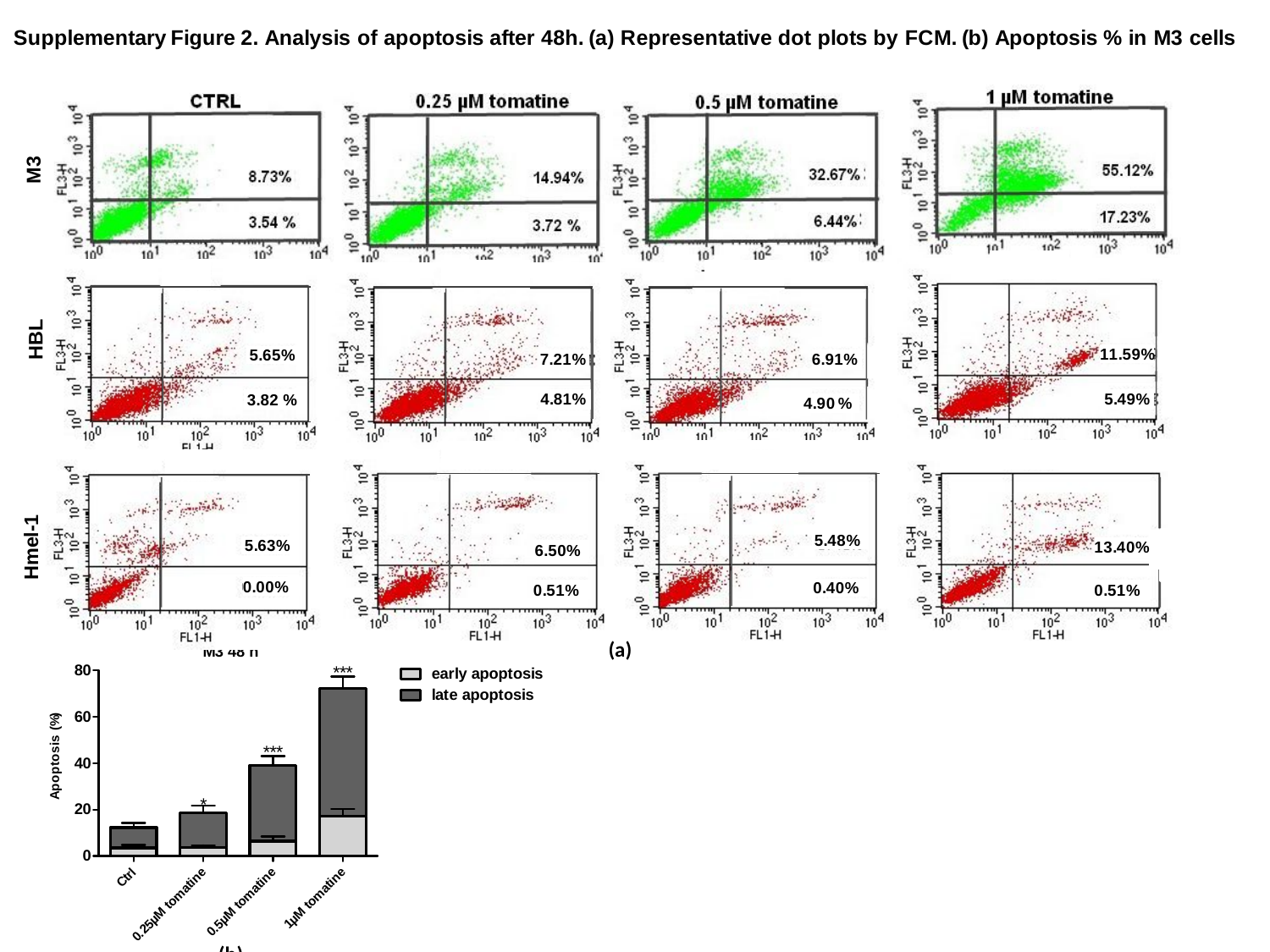

Supplement: Supplementary file 1 [file ijms-21-05243-s001.zip › suppl fig2 final.pptx]

## Slide 1
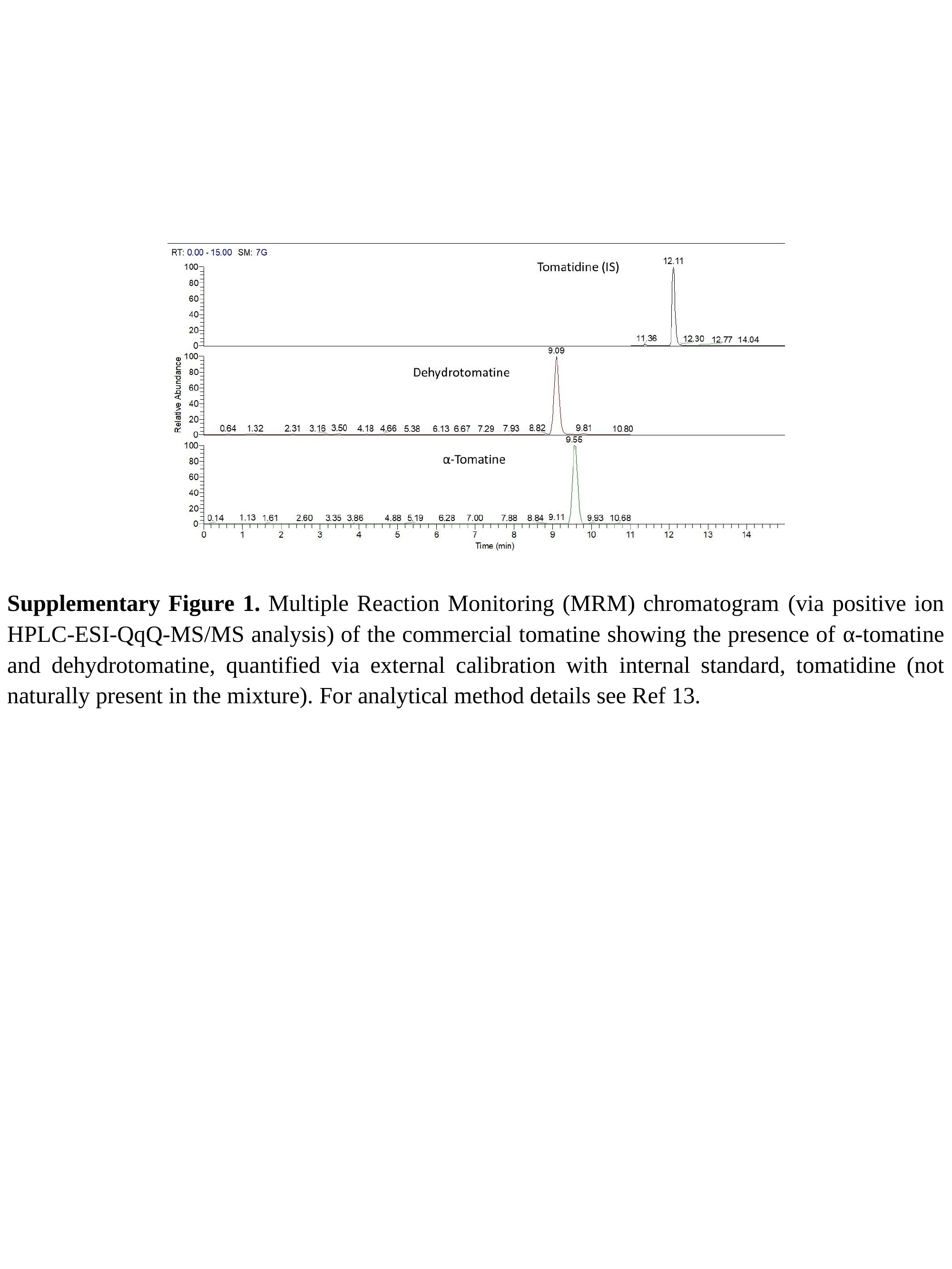

Supplement: Supplementary file 1 [file ijms-21-05243-s001.zip › suppl fig1 final.pptx]
